# Influence of Gender on Transcatheter Aortic Valve Implantation: A Systematic Review and Meta-Analysis

**DOI:** 10.31083/j.rcm2404116

**Published:** 2023-04-18

**Authors:** Zekun Lang, Youqi Zhu, Gaxue Jiang, Pengfei Ji, Xiaoqi Zhang, Yurong Zhang, Xince Sun, Ming Bai

**Affiliations:** ^1^The First Clinical Medical College of Lanzhou University, 730000 Lanzhou, Gansu, China; ^2^The First Hospital of Lanzhou University, Heart Center, Gansu Provincial Cardiovascular Disease Clinical Medical Research Center, Gansu Provincial Key Laboratory of Cardiovascular Disease, 730000 Lanzhou, Gansu, China; ^3^The Second Clinical Medical College of Lanzhou University, 730000 Lanzhou, Gansu, China

**Keywords:** transcatheter aortic valve implantation, sex characteristics, survival, meta-analysis

## Abstract

**Background:**

To assess whether there are differences in common postoperative complications and survival between men and women after transcatheter aortic valve implantation.

**Methods:**

We searched the Cochrane Library, PubMed, Embase, and the Web of Science from January 2000 to August 2022. Gender-related articles reporting complications and mortality after transcatheter aortic valve implantation were identified. The primary outcomes were the thirty-day mortality, one-year mortality and perivalvular leakage. The secondary outcomes were conversion to open heart surgery during operation, ejection fraction after operation, reintervention and other common postoperative complications. Data were pooled using the risk ratio or standardized mean difference with 95% confidence interval. Subgroup analysis, meta-regression, sensitivity analysis, egger’s test and begg’s test were performed. The original study protocol was registered prospectively with PROSPERO (CRD42021245858).

**Results:**

There were 24 studies, a total of 92,499 patients, enrolled in our systematic review and meta-analysis, including 43,948 men and 48,551 women. Comprehensive analysis showed significant differences in gender in postoperative complications and survival after transcatheter aortic valve implantation. Men had a significantly higher risk of perivalvular leakage (risk ratio (RR) = 1.42; 95% CI: 1.15 to 1.75; *p* = 0.001; $I^{2}$ = 68%), but lower risk in bleeding (RR = 0.69; 95% CI: 0.61 to 0.79; *p *< 0.00001; $I^{2}$ = 82%), vascular complications (RR = 0.56; 95% CI: 0.52 to 0.61; *p *< 0.00001; $I^{2}$ = 48%), and stroke (RR = 0.86; 95% CI: 0.80 to 0.93; *p *< 0.00001; $I^{2}$ = 12%). The thirty-day mortality of men is slightly lower than that of women (RR = 0.87; 95% CI: 0.81 to 0.93; *p* = 0.0001; $I^{2}$ = 47%), the difference in one-year mortality was also significant (RR = 1.20; 95% CI: 1.08 to 1.33; *p* = 0.0008; $I^{2}$ = 59%). Univariate meta-regression analyses showed that pulmonary hypertension is the major source of heterogeneity in bleeding.

**Conclusions:**

Men after transcatheter aortic valve implantation have a lower risk of related postoperative complications, but a higher risk of paravalvular leak and no advantage in medium-term survival.

## 1. Introduction

For patients with severe aortic valve stenosis (AS), surgical aortic valve replacement (SAVR) used to be the only treatment that could prolong life. Still, elderly patients are often contraindicated with surgery due to advanced age, weak physical fitness, or other diseases. The guideline also recommends that transcatheter aortic valve implantation (TAVI) be an effective treatment [1].

As devices have been evolved, we now have smaller delivery systems, retrievable valves, and more stable operating systems that allow us to do TAVI more safely and for patients with more complex underlying conditions. Multiple studies have shown that TAVI is significantly safer and more effective than accepted standard therapies in high-risk and inoperable AS patients [2, 3, 4, 5]. Women have been shown to have an increased risk of adverse events after SAVR [6, 7, 8, 9]. But for TAVI, being female was found to have certain advantages [10, 11, 12, 13, 14, 15]. However, previous trials of gender differences in TAVI patients have been limited in size. In addition, not all studies agree that TAVI may be more beneficial for women, with some finding no difference in outcomes or an increased risk of adverse events in women. Therefore, the study aims to assess gender differences in patients undergoing TAVI.

## 2. Methods

Our study was performed in accordance with the Preferred Reporting Items for Systematic Reviews and Meta-Analyses Protocols (PRISMA) statement [16]. This protocol has been registered on the International Prospective Systematic Reviews Registry database (CRD42021245858).

### 2.1 Data Sources and Search Strategy

Electronic databases, including the Cochrane Library, PubMed, Embase, and the Web of Science, were searched from January 2000 to Aug 2022. We focused on peer-reviewed publications of clinical trials. The following searched combination of keywords was used: [(Transcatheter Aortic Valve Replacement OR Transcatheter Aortic Valve Replacement OR Transcatheter Aortic Valve Implantation OR TAVR OR TAVI)] AND [(aorta OR aorta OR aortas OR aortae OR Femoral Artery OR Femoral Arteries OR Common Femoral Artery OR Common Femoral Arteries OR Internal Carotid Arteries OR Internal Carotid Arteries OR Internal Carotid Artery OR transapical OR transapical OR apical OR ventricular apex OR apex OR apex cordis OR cardiac apex)] AND[(Aortic Valve Stenosis OR Aortic Valve Stenoses OR Aortic Stenosis OR Aortic Valve Insufficiency OR Aortic Regurgitation OR Aortic Incompetence OR Aortic Valve Incompetence)] in the title/abstract. At the same time, try to collect all relevant literature and search references to supplement possible omissions. The detailed search strategy is presented in the **Supplementary Material** in the form of a word document.

### 2.2 Study Selection

Two reviewers (LZK and DHS) initially screened independently at the title and abstract level and retrieved all eligible full-text studies for further screening. Disagreements were resolved by discussion with BM and ZYQ. All trials that reported relevant outcomes in men and women after TAVI were considered. If they respectively reported the outcomes of prognosis and survival after TAVI in males and females, such as bleeding, vascular complications, 30-day mortality, etc. Studies were excluded if: (i) case reports, conference abstracts, comments, etc.; (ii) studies that did not report both male and female outcomes after TAVI; (iii) studies that could not find the full text; (iv) TAVI was used in combination with any other cardiac surgery.

### 2.3 Data Extraction and Quality Assessment

For each included study, data were extracted by one reviewer (JPF) and checked for accuracy and completeness by another reviewer (ZXQ). Any differences are resolved through discussion, if necessary, in consultation with a third reviewer (BM and ZYQ). First, we extracted gender, age, body mass index (BMI), and comorbidities (hypertension, pulmonary hypertension, etc.) for inclusion in the study. Secondly, for outcome measures, cardiovascular mortality, bleeding, vascular complication, and stroke etc. were pooled for analysis.

The quality of all studies was assessed using the Newcastle-Ottawa Scale (NOS) by two independent authors (LZK and DHS). Two authors conducted the quality assessment of each included studies from three items: selection bias, comparability bias, and exposure bias. There are evaluation items under each item, and each item is indicated by a star when appropriate. The highest score for the comparability bias item is two stars. Any discrepancies were resolved by consensus.

### 2.4 Outcomes and Definitions

The primary outcomes were the thirty-day mortality, one-year mortality and perivalvular leakage (PVL). PVL in this context refers to new onset aortic valve leak due to surgery. Thirty-day mortality and one-year mortality can reflect the short-term and medium-term survival of patients, respectively. We selected conversion to open heart surgery during operation, ejection fraction (EF) after operation, reintervention and other common postoperative complications including bleeding, vascular complication, myocardial infraction (MI) etc. as secondary outcomes to assess the prognosis in patients of different sexes after TAVI.

### 2.5 Statistical Analysis

All data were analyzed by Review Manager 5.4 (The Cochrane Collaboration, Copenhagen, Denmark) and Stata SE 16.0 (Stata Corporation, Texas, USA). The risk ratio (RR) with 95% confidence intervals (CI) were estimated for dichotomous data and standard mean difference (SMD) with 95% CI for continuous data, respectively. Heterogeneity was tested by the $I^{2}$ test and Q test. If *p *< 0.1 or $I^{2}$ > 50%, the heterogeneity test between the research results is statistically significant, random effects model analysis is used. If $I^{2}$ < 50%, the fixed-effect model was used for analysis [17]. If $I^{2}$ > 50%, severe heterogeneity will be considered. Subgroup and meta-regression analysis were conducted to explore the possible source of heterogeneity. Use funnel plot, Begg’s test and Egger’s test to assess the risk of publication bias of studies when there are at least 8 studies. A significance level of α = 0.05 was set for all analyses. Sensitivity analysis was used to assess whether the results were robust and also to assess sources of heterogeneity.

## 3. Results

### 3.1 Study Selection

**Fig. 1.**
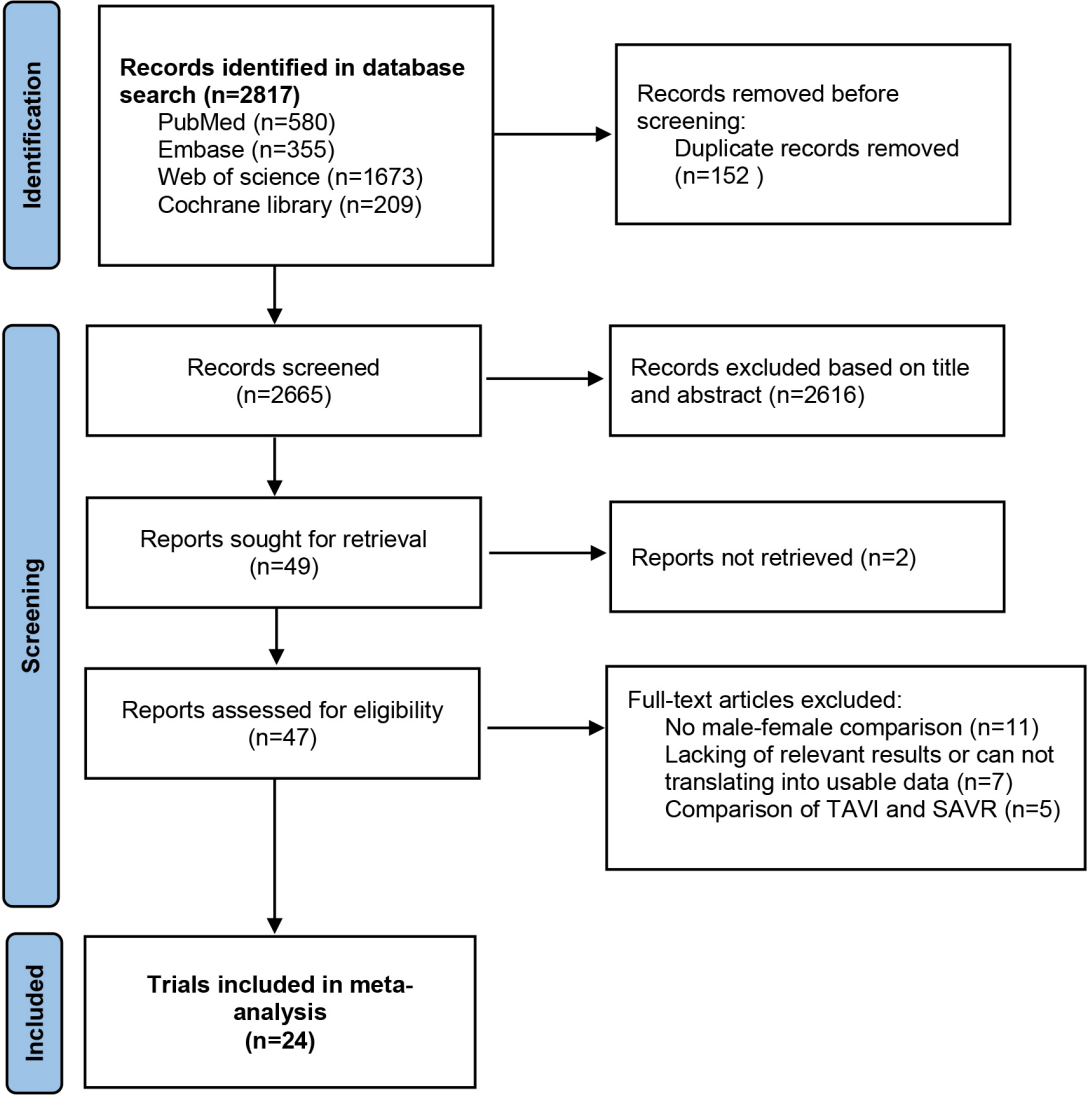
**Preferred reporting items for systematic reviews and meta-analyses (PRISMA) flowchart of selection**.

We retrieved 580 articles from Pubmed, 355 from embase, 209 from the cochrane library, and 1673 from web of science. A total of 2817 articles were retrieved from the database, and after deduplication, 2665 article titles and abstracts were evaluated. After initial title and abstract screening, 47 articles remained with downloaded full text. The authors evaluated the full text independently, and 24 eligible articles were included in the meta-analysis. Fig. 1 shows the whole process of literature retrieval and screening.

### 3.2 Study Characteristics and Quality Assessment

We sorted out the essential data characteristics of 24 included articles. The features of the included studies are shown in Tables 1,2 (Ref. [10, 11, 13, 14, 15, 18, 19, 20, 21, 22, 23, 24, 25, 26, 27, 28, 29, 30, 31, 32, 33, 34, 35, 36]). Due to the large number of articles included and the large sample size, some trials lacked the baseline data of some men and women. These trials were published between 2011 and 2022. Sample sizes for individual experiments ranged from 55 to 35,470. A total of 92,499 patients were involved, and the overall mean age of the patients included in the study was 81.7 years. Most of the studies used CoreValve and Edwards SAPIEN devices with prosthesis sizes of 23 mm–29 mm. Individual studies did not mention relevant conditions.

**Table 1.** **Baseline characteristics, medical conditions, and perioperative data of included studies for meta-analysis**.

Reference	Age (year)	MI (%)	DM (%)	HT (%)	CVA (%)	COPD (%)	PAD (%)	Stroke (%)	Dialysis (%)	PH (%)	PCI (%)
Ascenzo 2013 [18]	82.4	19.9	5.6	NA	NA	27.9	22.1	7.1	3.2	19.2	35.6
Biere 2015 [19]	82.8	NA	NA	NA	9.8	22.7	20.1	NA	NA	NA	56.9
Buchanan 2011 [20]	79.4	21.6	28.9	NA	15.7	38.0	NA	19.2	32.9	35.5	18.7
Buja 2013 [15]	81.0	22.0	26.0	75.0	NA	21.0	19.0	7.3	23.0	NA	29.0
Chandrasekhar 2016 [21]	82.0	NA	37.3	NA	NA	13.7	31.8	12.2	4.1	NA	35.7
Chang 2020 [22]	81.4	6.3	37.6	72.9	23.2	21.7	26.7	NA	7.7	NA	33.9
Czarnecki 2017 [23]	84.3	18.3	45.9	96.2	8.8	16.3	17.4	NA	2.6	57.4	30.2
Du 2020 [24]	74.3	1.4	17.8	49.3	NA	20.5	19.2	1.4	1.4	NA	11.0
Forrest 2016 [25]	83.3	27.6	37.6	92.7	NA	33.8	45.4	13.2	12.0	NA	38.1
Hayashida 2012 [14]	83.1	14.6	23.5	70.8	12.7	37.3	33.5	NA	NA	28.8	30.4
Humphries 2012 [13]	81.7	40.4	30.7	78.9	18.4	26.5	31.8	NA	2.5	NA	NA
Kaier 2018 [26]	80.9	6.6	33.4	62.5	NA	14.9	10.9	NA	NA	21.6	NA
Katz 2016 [27]	81.5	14.0	31.0	75.0	NA	19.0	17.0	8.0	NA	22.0	NA
Kodali 2016 [28]	84.5	26.1	36.6	91.8	26.2	44.6	42.8	NA	16.4	39.0	39.8
Madershahian 2014 [29]	82.4	NA	36.4	87.3	NA	NA	41.9	NA	NA	NA	NA8
Onorati 2014 [30]	81.9	2.8	24.7	NA	NA	28.8	22.2	NA	1.7	NA	25.1
Sherif 2014 [10]	81.7	15.9	34.4	NA	NA	24.0	30.7	8.0	NA	66.3	34.6
Singh 2019 [31]	72.9	NA	21.7	53.1	NA	NA	NA	NA	NA	NA	8.5
Stangl 2012 [32]	79.0	15.0	45.0	NA	NA	34.0	29.0	10.0	NA	56.0	41.0
Szerlip 2016 [33]	NA	NA	NA	NA	NA	NA	NA	NA	NA	NA	NA
Van Mieghem 2020 [34]	79.8	NA	34.5	NA	16.9	34.5	30.4	6.9	NA	NA	21.3
Vlastra 2019 [35]	82.3	14.0	31.0	79.0	NA	NA	15.0	10.0	NA	NA	22.0
Williams 2014 [11]	83.6	26.6	42.3	NA	NA	27.4	43.2	NA	NA	50.3	33.6
Wohrle 2022 [36]	80.6	NA	28.6	NA	NA	12.0	10.0	4.6	NA	NA	NA

MI, myocardial infarction; DM, diabetes mellitus; HT, hypertension; CVA, cerebrovascular accident; COPD, chronic obstructive pulmonary disease; PAD, peripheral artery disease; PH, pulmonary hypertension; PCI, percutaneous coronary intervention; NA, not available. Value are as mean.

**Table 2.** **Baseline characteristic**.

Reference	Country	Sex	N	Mean age (year)	BSA ($m^{2}$)	BMI (Kg/$m^{2}$)	LVEF (%)	Surgical approach
Ascenzo 2013 [18]	Italy	Male	161	81.65 ± 5.32	1.82 ± 0.14	NA	49.22 ± 13.5	Mainly transfemoral
		Female	216	82.90 ± 5.45	1.65 ± 0.19	NA	54.70 ± 11.3	
Biere 2015 [19]	France	Male	2005	81.6 ± 7.5	NA	26.3 ± 4.5	50.1 ± 14.3	Mainly transfemoral
		Female	1967	84.0 ± 6.6	NA	25.7 ± 5.4	56.6 ± 13.3	
Buchanan 2011 [20]	Italy	Male	159	78.8 ± 7.8	1.84 ± 0.16	25.9 ± 4.1	50.8 ± 12.9	Mainly transfemoral
		Female	146	80.1 ± 6.8	1.70 ± 0.16	26.6 ± 4.9	52.2 ± 12.6	
Buja 2013 [15]	Italy	Male	291	80 ± 7	1.8 ± 0.2	NA	49 ± 13	Mainly transfemoral
		Female	368	82 ± 5	1.7 ± 0.2	NA	53 ± 13	
Chandrasekhar 2016 [21]	Australia	Male	11,844	81.67 ± 8.63	1.9 (1.8, 2.1)	27.51 ± 5.68	50.6 ± 14.3	Transfemoral and transapical
		Female	11,808	82.28 ± 8.52	1.7 (1.6, 1.9)	28.38 ± 7.48	56.7 ± 12.5	
Chang 2020 [22]	China	Male	96	81.7 ± 8.9	1.7 ± 0.2	24.2 ± 3.9	53.4 ± 10.9	Mainly transfemoral
		Female	125	81.2 ± 8.0	1.5 ± 0.2	24.5 ± 4.4	56.5 ± 9.4	
Czarnecki 2017 [23]	Canada	Male	546	83 (77, 87)	NA	NA	NA	Transfemoral and transapical
		Female	453	85 (80, 88)	NA	NA	NA	
Du 2020 [24]	China	Male	36	74.92 ± 6.87	1.7 ± 0.2	22.04 ± 2.76	52.0 ± 14.1	NA
		Female	37	73.70 ± 5.38	1.6 ± 0.1	23.26 ± 3.20	55.5 ± 14.9	
Forrest 2016 [25]	America	Male	1979	82.7 ± 7.9	NA	NA	51.0 ± 14.3	NA
		Female	1708	84.0 ± 7.6	NA	NA	57.7 ± 12.3	
Hayashida 2012 [14]	France	Male	129	82.4 ± 6.5	1.85 ± 0.16	25.9 ± 4.1	47.2 ± 14.6	Transfemoral and transapical
		Female	131	83.8 ± 5.9	1.65 ± 0.17	25.6 ± 4.8	53.5 ± 12.9	
Humphries 2012 [13]	Canada	Male	312	82 (76, 86)	1.9 (1.8, 2.0)	NA	55 (40, 60)	Transfemoral and transapical
		Female	329	83 (76, 87)	1.6 (1.5, 1.8)	NA	60 (55, 65)	
Kaier 2018 [26]	Germany	Male	16,126	73.7	NA	NA	NA	Transfemoral and transapical
		Female	19,344	78.0	NA	NA	NA	
Katz 2016 [27]	Brazil	Male	401	80.6 ± 7.5	NA	26.0 ± 4.0	55.4 ± 15.6	Mainly transfemoral
		Female	418	82.4 ± 7.0	NA	26.6 ± 5.4	61.9 ± 13.4	
Kodali 2016 [28]	America	Male	1339	84.1 ± 7.3	1.93 ± 0.20	NA	55.5 ± 11.9	Transfemoral and transapical
		Female	1220	84.9 ± 6.9	1.66 ± 0.21	NA	49.5 ± 13.3	
Madershahian 2014 [29]	Germany	Male	26	80.6 ± 7.1	1.8 ± 0.18	NA	52.6 ± 12.0	NA
		Female	29	84.0 ± 5.6	1.9 ± 0.20	NA	59.5 ± 15.4	
Onorati 2014 [30]	Italy	Male	297	81.1 ± 6.6	NA	NA	48.2 ± 12.6	Mainly transfemoral
		Female	428	82.4 ± 6.3	NA	NA	53.1 ± 11.4	
Sherif 2014 [10]	Germany	Male	605	80.3 ± 6.4	NA	NA	49 ± 15	Mainly transfemoral
		Female	827	82.8 ± 5.8	NA	NA	56 ± 14	
Singh 2019 [31]	England	Male	425	71.8 ± 10.5	NA	27.8 ± 4.6	59.3 ± 11.9	NA
		Female	249	74.9 ± 10.7	NA	27.2 ± 5.8	61.3 ± 11.9	
Stangl 2012 [32]	Germany	Male	42	77 ± 9	2.0 ± 0.2	27 ± 5	46.7 ± 14.8	Mainly transfemoral
		Female	58	80 ± 8	1.7 ± 0.2	26 ± 6	54.3 ± 8.4	
Szerlip 2016 [33]	America	Male	338	NA	NA	NA	NA	NA
		Female	245	NA	NA	NA	NA	
Van Mieghem 2020 [34]	Netherlands	Male	498	79.6 ± 6.4	2.0 ± 0.2	NA	NA	NA
		Female	366	80.0 ± 5.9	1.8 ± 0.2	NA	NA	
Vlastra 2019 [35]	Netherlands	Male	5261	82 (77, 85)	NA	27.1 ± 4.1	NA	NA
		Female	7120	83 (79, 86)	NA	27.3 ± 5.4	NA	
Williams 2014 [11]	America	Male	201	82.9 ± 7.11	1.93 ± 0.21	NA	49.6 ± 14.4	Transfemoral and transapical
		Female	146	84.5 ± 6.34	1.69 ± 0.23	NA	55.2 ± 18.6	
Wohrle 2022 [36]	Germany	Male	831	80.04 ± 6.93	NA	28.16 ± 4.95	NA	NA
		Female	813	81.12 ± 6.16	NA	28.11 ± 6.23	NA	

N, number; BSA, body surface area; BMI, body mass index; LVEF, left ventricular ejection fraction; NA, not available. Value are as mean, mean ± SD or median (Q1, Q3).

After the evaluation of quality, we found that all included studies were on the upper-middle quality by NOS. Each has a score greater than five stars and meets the criteria for inclusion in the meta-analysis. The final result is shown in Table 3.

**Table 3.** **Results of NOS quality assessment**.

Number	First author	Published year	Selection	Comparability	Outcome	Score
1	Ascenzo	2013	★★★★	★★	★★	8
2	Biere	2015	★★★★	★★	★★	8
3	Buchanan	2011	★★★	★★	★★	7
4	Buja	2013	★★★★	★★	★★	8
5	Chandrasekhar	2016	★★★	★	★★	6
6	Chang	2020	★★★★	★★	★★	8
7	Czarnecki	2017	★★★	★	★★	6
8	Du	2020	★★★★	★	★★	7
9	Forrest	2016	★★★★	★★	★	7
10	Hayashida	2012	★★★★	★	★★	7
11	Humphries	2012	★★★★	★	★★	7
12	Kaier	2018	★★★★	★★	★★	8
13	Katz	2016	★★★★	★★	★★	8
14	Kodali	2016	★★★	★★	★	7
15	Madershahian	2014	★★★★	★	★	6
16	Onorati	2014	★★★★	★	★★	7
17	Sherif	2014	★★★★	★	★	6
18	Singh	2019	★★★★	★★	★★	8
19	Stangl	2012	★★★	★★	★★	7
20	Szerlip	2016	★★★	★★	★	6
21	Van Mieghem	2020	★★★★	★★	★★	8
22	Vlastra	2019	★★★	★★	★★	7
23	Williams	2014	★★★★	★	★★	7
24	Wohrle	2022	★★★★	★★	★	7

### 3.3 Main Outcomes of Study Results

**Fig. 2.**
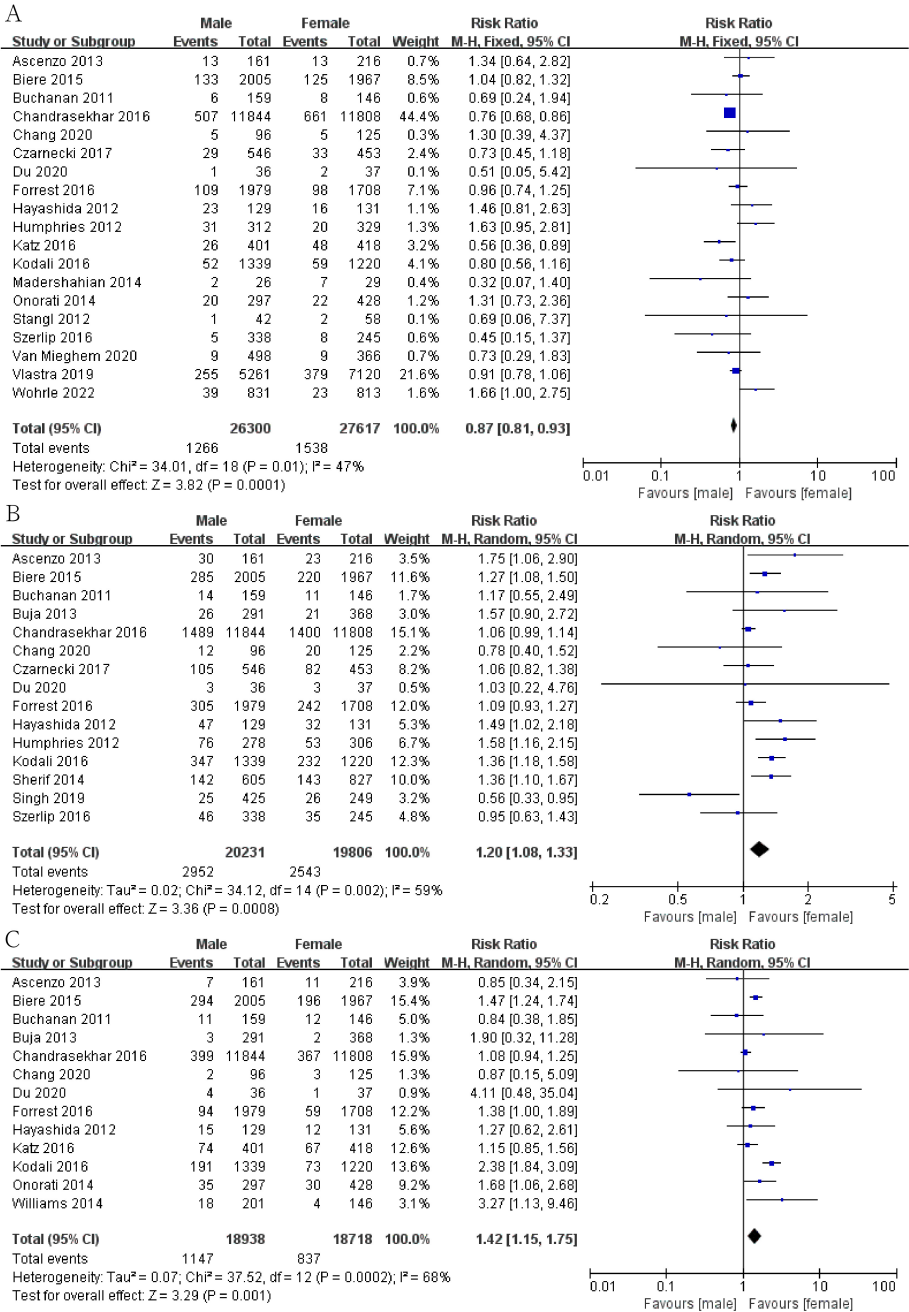
**Forest plot of (A) thirty-day mortality**. (B) One-year mortality. (C) Perivalvular leakage (PVL).

**Fig. 3.**
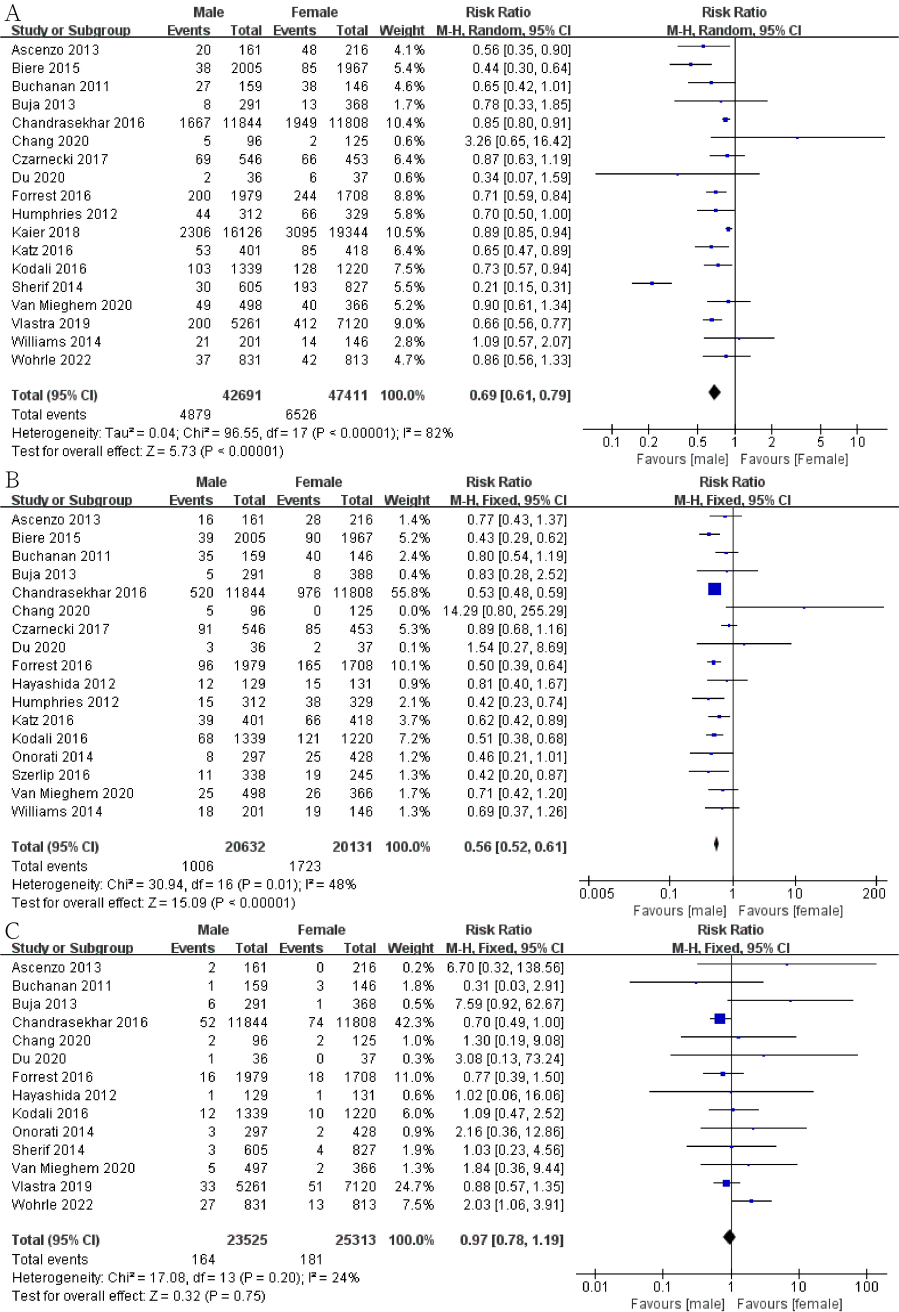
**Forest plot of (A) stroke**. (B) Kidney injury. (C) Conversion to open heart surgery.

**Fig. 4.**
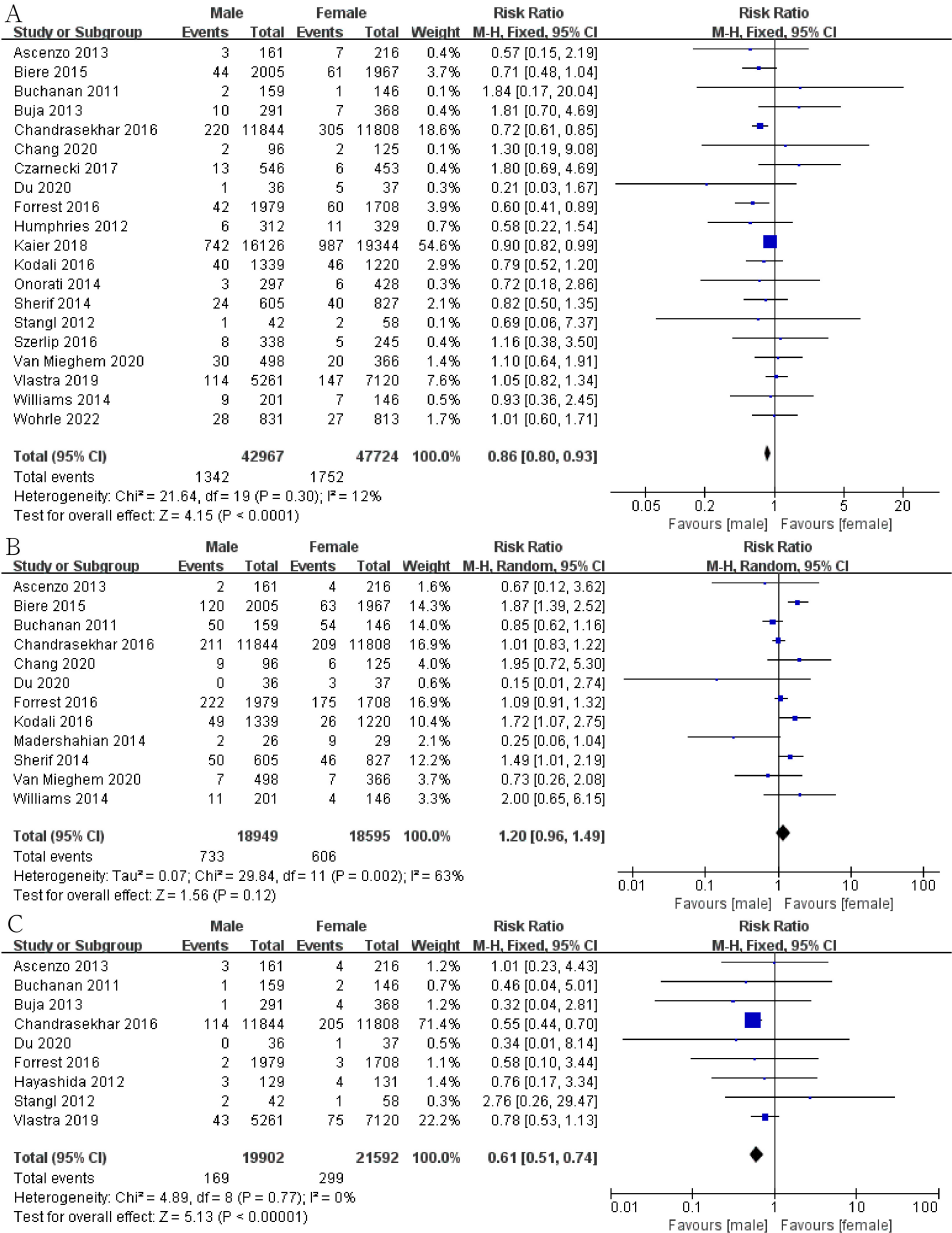
**Forest plot of (A) reintervention**. (B) Atrial fibrillation. (C) Ejection fraction (EF).

The results show that men had a lower risk of death at thirty days (Fig. 2A; RR, 0.87; 95% CI, 0.81 to 0.93; *p* = 0.0001; $I^{2}$ = 47%) and a higher risk of death at one year (Fig. 2B; RR, 1.20; 95% CI, 1.08 to 1.33; *p* = 0.0008; $I^{2}$ = 59%). Meanwhile, men have a higher risk of PVL (Fig. 2C; RR, 1.42; 95% CI, 1.15 to 1.75; *p* = 0.001; $I^{2}$ = 68%). In addition, men had a lower risk of intraoperative conversion to open heart surgery (Fig. 3C; RR, 0.61; 95% CI, 0.51 to 0.74; *p *< 0.00001; $I^{2}$ = 0) and had a lower postoperative EF (Fig. 4C; SMD, –0.42; 95% CI, –0.48 to –0.37; *p *< 0.00001; $I^{2}$ = 25%) than women.

**Fig. 5.**
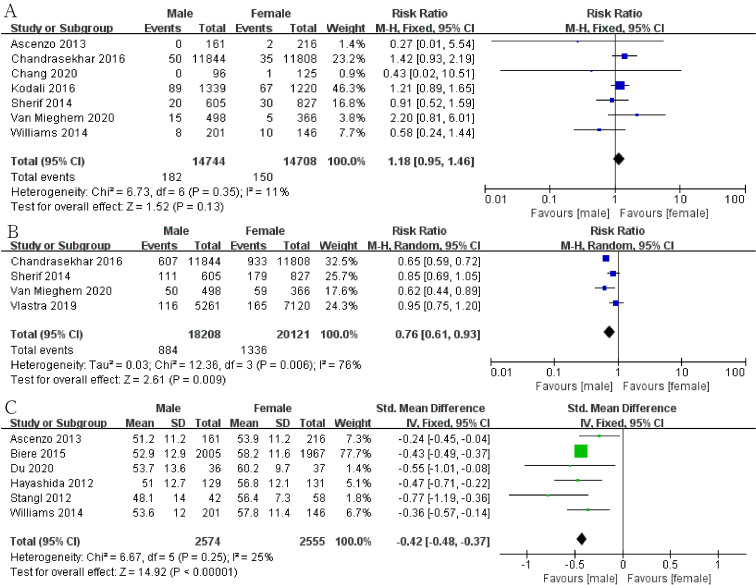
**Forest plot of (A) bleeding**. (B) Vascular complication. (C) Myocardial infraction (MI).

We explored the risk of stroke, bleeding, vascular complications, atrial fibrillation kidney injury and MI to evaluate the prognosis of patients after TAVI. The risk of stroke in male group was lower than that in female group (Fig. 3A; RR, 0.86; 95% CI, 0.80 to 0.93; *p* = 0.0001; $I^{2}$ = 12%). In the statistics of 90,691 patients, we found that men have a lower risk of reintervention (Fig. 4A; RR, 0.86; 95% CI, 0.80 to 0.93; *p *< 0.0001; $I^{2}$ = 12%). The risk of major bleeding in male group was significantly lower than that in the female group (Fig. 5A; RR, 0.69; 95% CI, 0.61 to 0.79; *p *< 0.00001; $I^{2}$ = 82%). As for major vascular complications, we analyzed data from 20,632 male patients and 20,131 female patients. The risk of major vascular complications in male group was also significantly lower than that in the female group (Fig. 5B; RR, 0.56; 95% CI, 0.52 to 0.61; *p *< 0.00001; $I^{2}$ = 48%). We also found that men had a lower risk of atrial fibrillation after surgery (Fig. 4B; RR, 0.76; 95% CI, 0.61 to 0.93; *p* = 0.009; $I^{2}$ = 76%). However, no significant gender differences were shown in postoperative MI (Fig. 5C) and kidney injury (Fig. 3B).

### 3.4 Subgroup Analysis

**Fig. 6.**
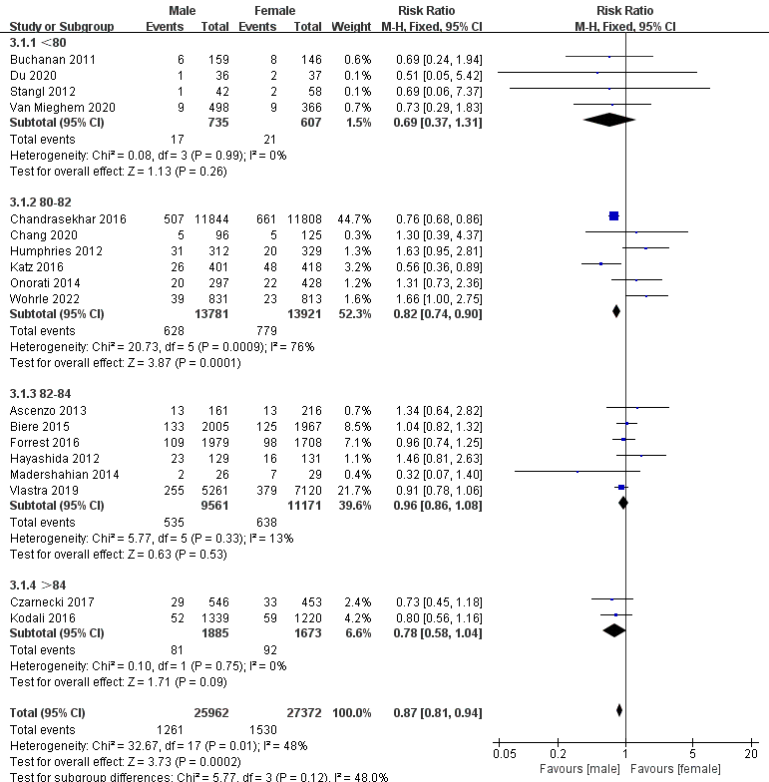
**Subgroup analysis for different ages in thirty-day mortality**.

**Fig. 7.**
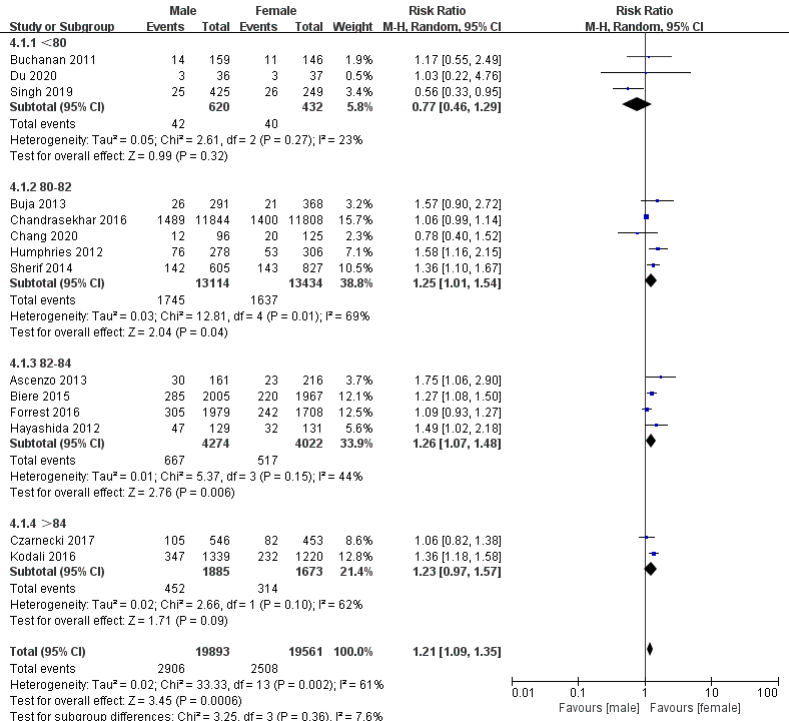
**Subgroup analysis for different ages in one-year mortality**.

**Fig. 8.**
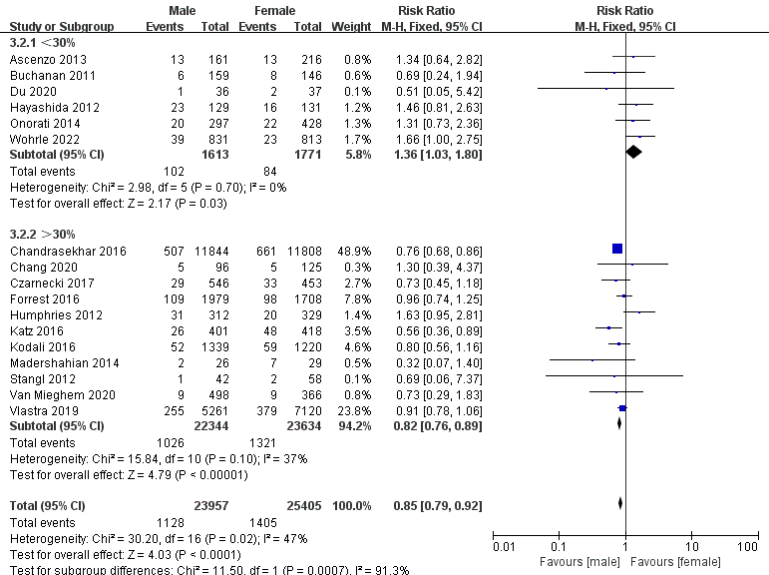
**Subgroup analysis for DM in thirty-day mortality**. DM, diabetes mellitus.

**Fig. 9.**
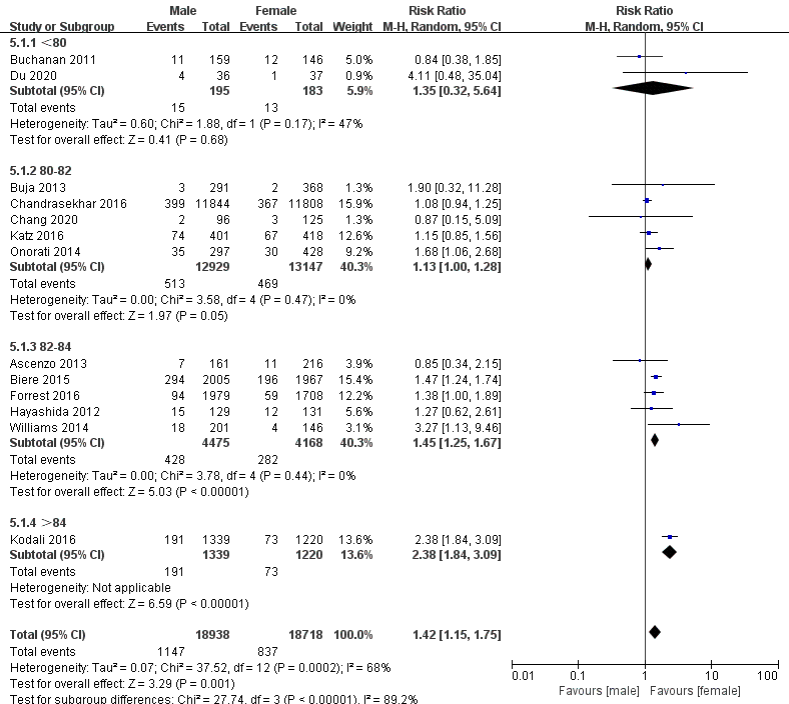
**Subgroup analysis for age in Perivalvular leakage**.

We performed subgroup analyses of thirty-day mortality and one-year mortality according to different age groups, different proportions of patients with hypertension (HT) and diabetes mellitus (DM). The results show that among patients younger than 80 years old, women had a higher 30-day mortality risk (RR, 0.69), while there was no significant difference in the 30-day mortality risk between women and men among patients aged 82–84 years (RR, 0.96). Meanwhile, age was the main source of heterogeneity in thirty-day mortality (Fig. 6). Conversely, the subgroup analysis of one-year mortality found that women had a higher risk of death among patients under the age of 80, and men over the age of 80 had a higher risk of death (Fig. 7). A subgroup analysis of DM also showed an interesting result. Women with a lower prevalence of DM had a lower thirty-day risk of death, while those with a higher prevalence of DM had a higher thirty-day risk of death. And DM may be one of the major sources of heterogeneity in thirty-day mortality (Fig. 8). The subgroup analysis of the risk of PVL in different age groups showed that there was no significant difference in the risk of PVL among different age groups, but age was one of the sources of heterogeneity in PVL (Fig. 9). Subgroup analyses on hypertension were not statistically and clinically significant, and the results are presented in the **Supplementary Material**.

We also performed subgroup analyses of PVL by different age groups, different proportions of patients with peripheral artery disease (PAD). The results show that the risk of PVL was higher in patients older than 84 years, twice as high as in women. Also, age is one of the main sources of PVL heterogeneity.

### 3.5 Meta-Regression for the Potential Sources of Heterogeneity

Age, DM, HT, PAD, pulmonary hypertension (PH) and percutaneous coronary intervention (PCI) were included in the random-effect univariate meta-regression analyses for bleeding. Results suggest PH as a possible source of heterogeneity. We also performed a meta-regression analysis of one-year mortality and PVL, including age, myocardial infarction (MI), DM, HT, chronic obstructive pulmonary disease (COPD), PAD, stroke, diagnosis, PH, PCI and other variables, but major sources of heterogeneity were not found. Details are shown in **Supplementary Material**.

### 3.6 Publication Bias Assessment and Sensitivity Analysis

The funnel plot of bleeding, vascular complications, stroke, PVL, thirty-day mortality and one-year mortality has no obvious publication bias, the details were shown in **Supplementary Material**. Begg’s test and egger’s test also showed that there was no obvious publication bias in bleeding, vascular complications, stroke, PVL, conversion to open heart surgery, one-year mortality and thirty-day mortality. Detailed results are presented in **Supplementary Material**. For other outcomes, due to the small number of included studies, no begg’s test or egger’s test was performed. Sensitivity analysis shows that the combined results are robust and reliable. The results of the sensitivity analysis are presented in the **Supplementary Material**.

## 4. Discussion

The main results of this meta-analysis show that after TAVI, men have an advantage in short-term survival, whereas women have an advantage in medium-term survival outcomes. The main common complications after TAVI include PVL, bleeding, vascular complications, etc. Women are at higher risk than men for postoperative bleeding, vascular complications, stroke, and atrial fibrillation. However, the risk of postoperative PVL in women is significantly lower than that in men. Overall, men after TAVI have a lower risk of related postoperative complications and an advantage in short-term survival.

PVL is a unique complication after valve replacement and is a common reason for reoperation after valve replacement due to its lack of tendency to close spontaneously. The lower risk of PVL in women is most likely related to the smaller diameter of the prosthesis used by women [37, 38]. Stroke as a major common complication of the nervous system can reflect the prognosis of patients after surgery. The study by Kaier *et al*. [26] found that women have a greater risk of postoperative stroke, which is consistent with our finding. Bleeding is one of the most common complications of TAVI, and vascular complications are also important complications after cardiovascular surgery. Stangl *et al*. [39] showed a 1.72-fold increased incidence of major vascular complications in women. And significantly lower risk of bleeding in men was found in the study by Zhao *et al*. [40] Survival of TAVI patients has been a focus of research, and Pighi *et al*. [41] found that female gender was a significant predictor of thirty-day mortality risk, but not for one-year mortality. This also verifies the reliability of the results of our meta-analysis of survival data on the other hand.

Women are the main risk factors for major vascular complications, major bleeding, and stroke, and their short-term survival rate is also low, but their prognosis is better. The possible reasons are as follows: ① First, major bleeding, stroke, and vascular complications are not the leading causes of death. Therefore, high-risk factors have no direct impact on women’s medium-term mortality. Meanwhile, previous studies by Amabile *et al*. [42] pointed out that vascular complications have no significant effect on TAVI prognosis. ② According to Chiam *et al*. [43], women are generally shorter than men, the aortic annulus is smaller, and mild or more paravalvular leakage also occurs less in women. Another study by Chiam *et al*. [44]. showed that female patients had better left ventricular ejection fractions (LVEF) and fewer complications in coronary heart disease, smoking, and chronic lung disease. Conversely, diabetes, chronic obstructive pulmonary disease, and atherosclerotic conditions are more common among men. These factors will undoubtedly increase the burden of male prognosis. ③ From the perspective of pathophysiology, the difference between men and women can also explain this problem. The expression of collagen I, collagen III, metal matrix proteinase-2, and metal matrix proteinase-9 decreased in female patients with aortic stenosis, resulting in a lower degree of cardiac remodeling and fibrosis in female patients with aortic stenosis than in male patients with aortic stenosis [45, 46, 47], making it easier for the valve membranes of female patients to adhere to each other and thicken and harden the valves [48].

We noticed mild heterogeneity in stroke ($I^{2}$ = 12%), vascular complication ($I^{2}$ = 48%), postoperative EF ($I^{2}$ = 25%) and conversion to open heart surgery ($I^{2}$ = 0). We believe that the heterogeneity is low, which is not explained. However, we analyzed heterogeneity for bleeding ($I^{2}$ = 82%), PVL ($I^{2}$ = 68%), atrial fibrillation ($I^{2}$ = 76%), thirty-day mortality ($I^{2}$ = 47%) and one-year mortality ($I^{2}$ = 59%). On this basis, the heterogeneity was studied using subgroup analysis and meta-regression. The main sources of heterogeneity were age, diabetes, and pulmonary hypertension, respectively. Apart from this, the slight heterogeneity may be related to the internal factors of each survey and may also be related to other comorbid diseases besides the cardiovascular system of the selected patients. In addition, significant heterogeneity was found in the analysis of bleeding and atrial fibrillation ($I^{2}$ = 82%, 76%, respectively). But the heterogeneity significantly reduced ($I^{2}$ = 63%, 48%, respectively) when we respectively excluded Sherif 2014 [10] and Chandrasekhar 2016 [21] for the combined analysis of the risk ratio of significant bleeding and major vascular complications. These two documents are not high-standard articles in our quality evaluation, so the differences in heterogeneity are likely to be related to their internal factors. Age is one of the main internal factors. TAVI surgery itself is designed for patients who are too old to undergo surgical valve replacement, so patients are often older, have many comorbidities, and have poor general conditions. The elderly also has poor tolerance to surgery and anesthesia. Many of the above factors may have adverse effects on the prognosis of patients, so age is also likely to be one of the main sources of potential heterogeneity. In addition to the above two documents, arbitrarily deleting the documents in the research will not affect the research results, which means that the results of our analysis are robust and reliable.

Our meta-analysis has several limitations: (i) Although great care was taken to include the study, the possibility of data duplication due to overlap in the selected patients cannot be completely ruled out. (ii) Because the individual baseline data in the included literature are indeed and the baseline data are not uniform, we cannot analyze the baseline data of all patients in the sample. (iii) Due to the different follow-up times of each study, it is impossible to obtain long-term survival data for analysis. (iiii) Due to differences in countries and years of inclusion in the trials, we were unable to determine the potential effect of different devices and different ages on outcomes for women and men [49].

## 5. Conclusions

In conclusion, men have a lower risk of bleeding, vascular complications, atrial fibrillation and stroke after TAVI, but men are at higher risk for postoperative PVL. In addition, men have an advantage in short-term survival, while women have an advantage in medium-term survival. Studies on heterogeneity suggest that age and diabetes may be important prognostic factors for TAVI.

## Supplementary Material

Supplementary material associated with this article can be found, in the online version, at https://doi.org/10.31083/j.rcm2404116.
