# Supplementary figures and images for: Influence of Gender on Transcatheter Aortic Valve Implantation: A Systematic Review and Meta-Analysis
Source: Rev Cardiovasc Med. 2023 Apr 18;24(4):116. doi: 10.31083/j.rcm2404116 (PMC11273049; doi:10.31083/j.rcm2404116)

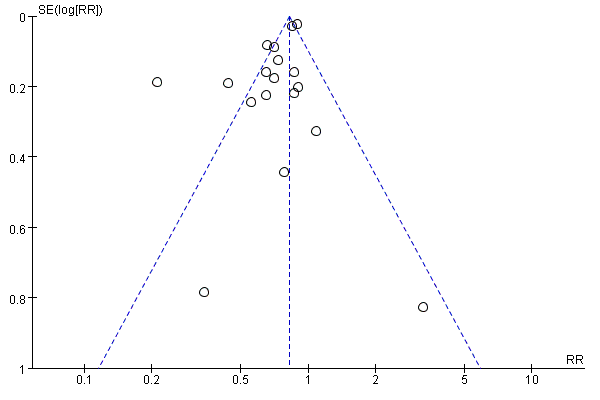

Supplement: Supplementary file 1 [file 2153-8174-24-4-116-s1.zip › Funnel plot of bleeding.png]

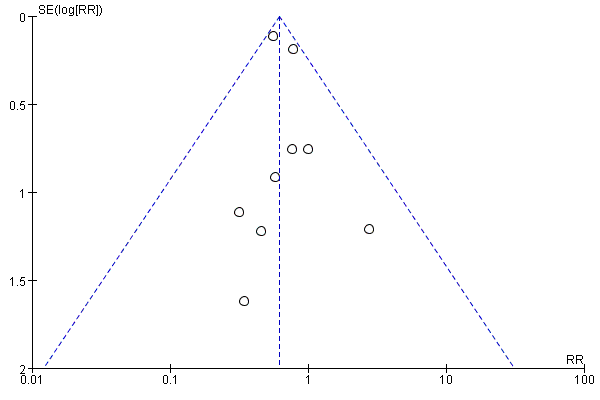

Supplement: Supplementary file 1 [file 2153-8174-24-4-116-s1.zip › Funnel plot of conversion to open heart surgery.png]

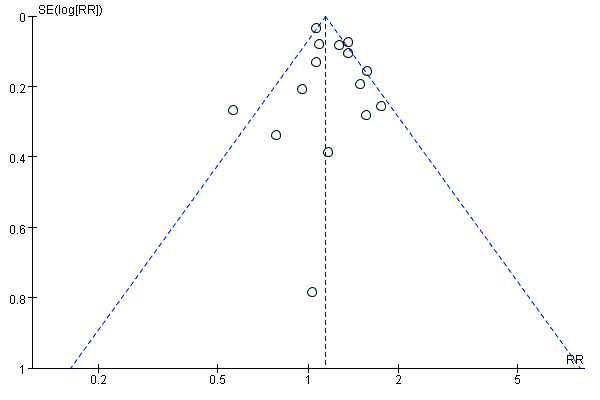

Supplement: Supplementary file 1 [file 2153-8174-24-4-116-s1.zip › Funnel plot of one-year mortality.png]

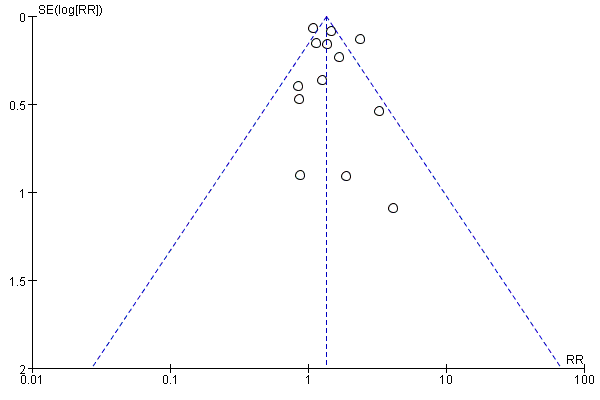

Supplement: Supplementary file 1 [file 2153-8174-24-4-116-s1.zip › Funnel plot of PVL.png]

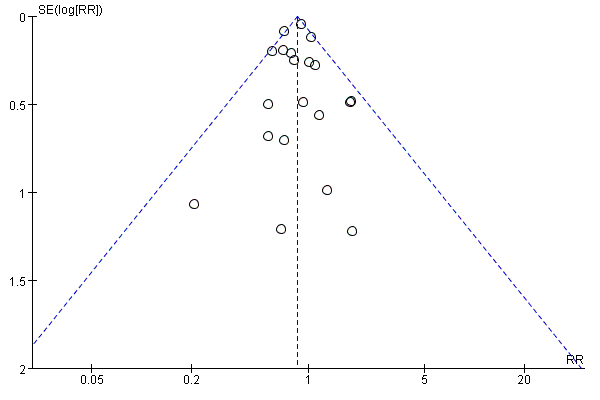

Supplement: Supplementary file 1 [file 2153-8174-24-4-116-s1.zip › Funnel plot of stroke.png]

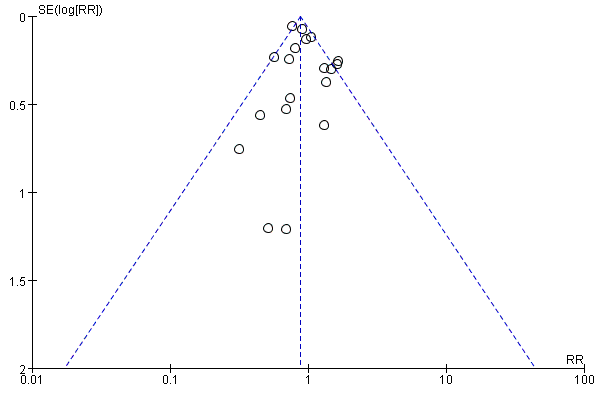

Supplement: Supplementary file 1 [file 2153-8174-24-4-116-s1.zip › Funnel plot of thirty-day mortality.png]

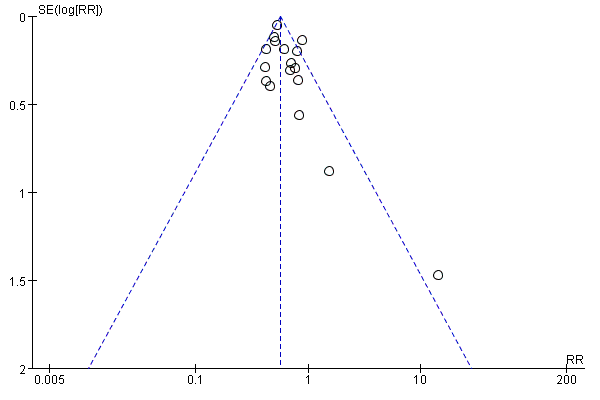

Supplement: Supplementary file 1 [file 2153-8174-24-4-116-s1.zip › Funnel plot of vascular complication.png]

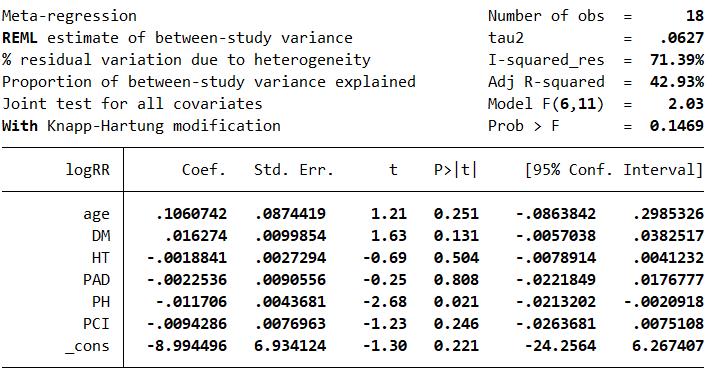

Supplement: Supplementary file 1 [file 2153-8174-24-4-116-s1.zip › meta regression of bleeding.jpg]

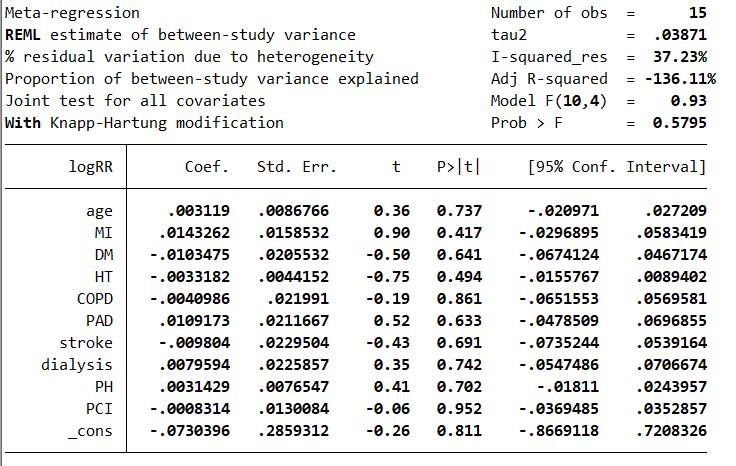

Supplement: Supplementary file 1 [file 2153-8174-24-4-116-s1.zip › meta regression of one-year mortality.jpg]

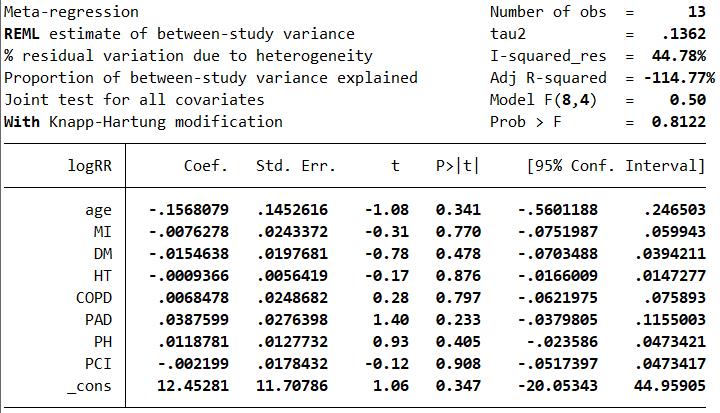

Supplement: Supplementary file 1 [file 2153-8174-24-4-116-s1.zip › meta regression of PVL.jpg]

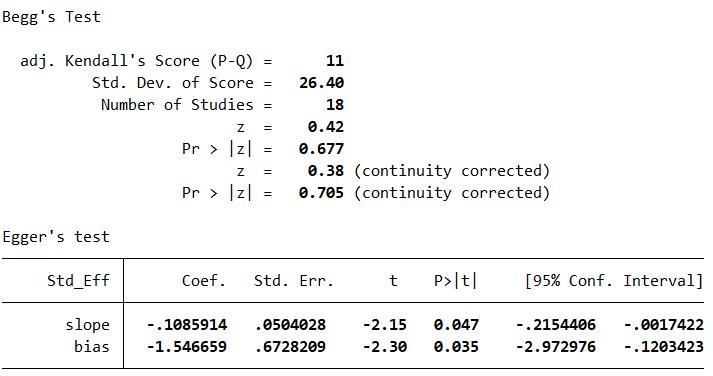

Supplement: Supplementary file 1 [file 2153-8174-24-4-116-s1.zip › publication bias of bleeding.jpg]

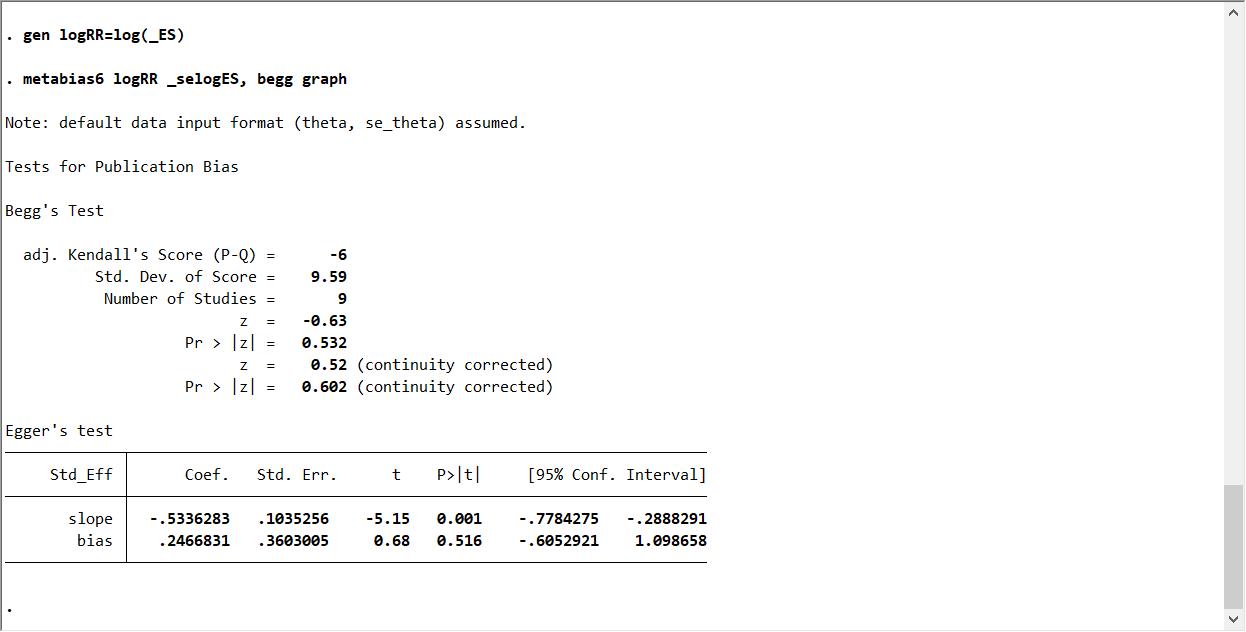

Supplement: Supplementary file 1 [file 2153-8174-24-4-116-s1.zip › publication bias of conversion to open surgery.jpg]

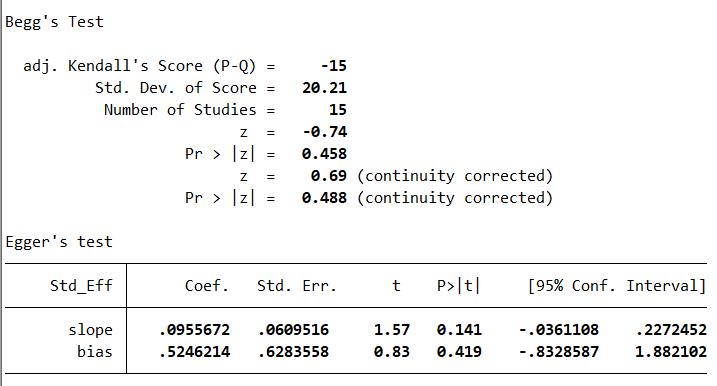

Supplement: Supplementary file 1 [file 2153-8174-24-4-116-s1.zip › publication bias of one-year mortality.jpg]

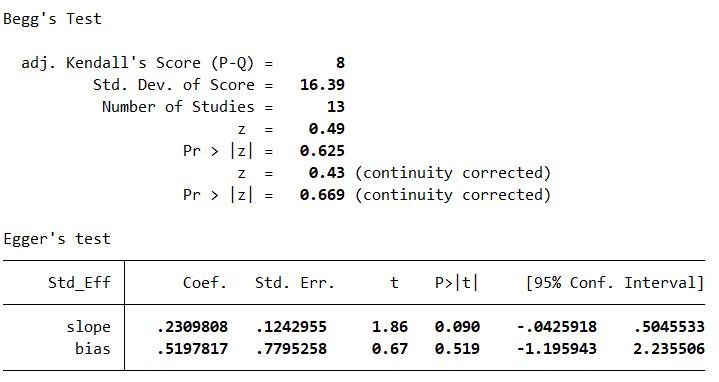

Supplement: Supplementary file 1 [file 2153-8174-24-4-116-s1.zip › publication bias of PVL.jpg]

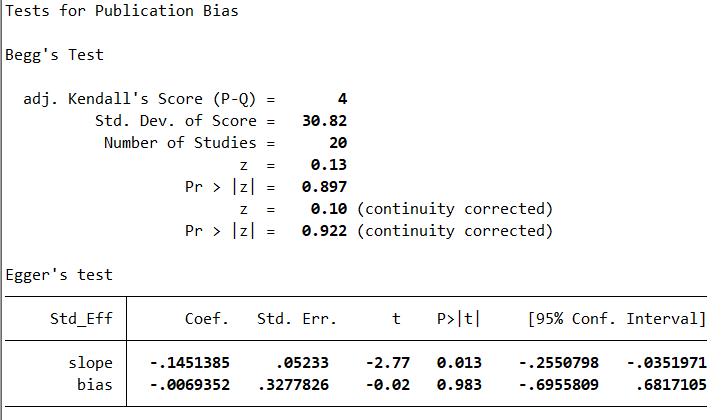

Supplement: Supplementary file 1 [file 2153-8174-24-4-116-s1.zip › publication bias of stroke.jpg]

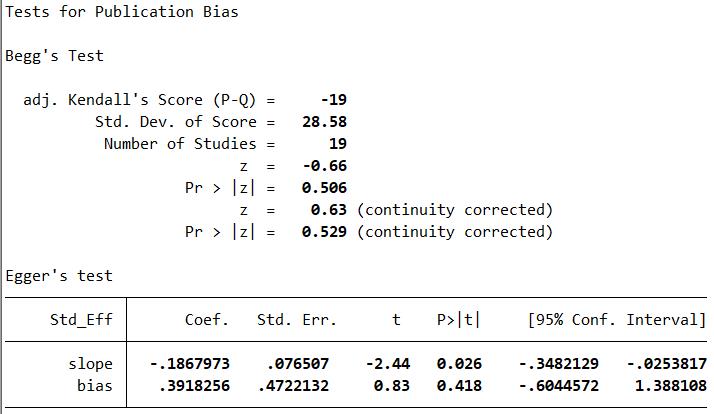

Supplement: Supplementary file 1 [file 2153-8174-24-4-116-s1.zip › publication bias of thirty-day mortality.jpg]

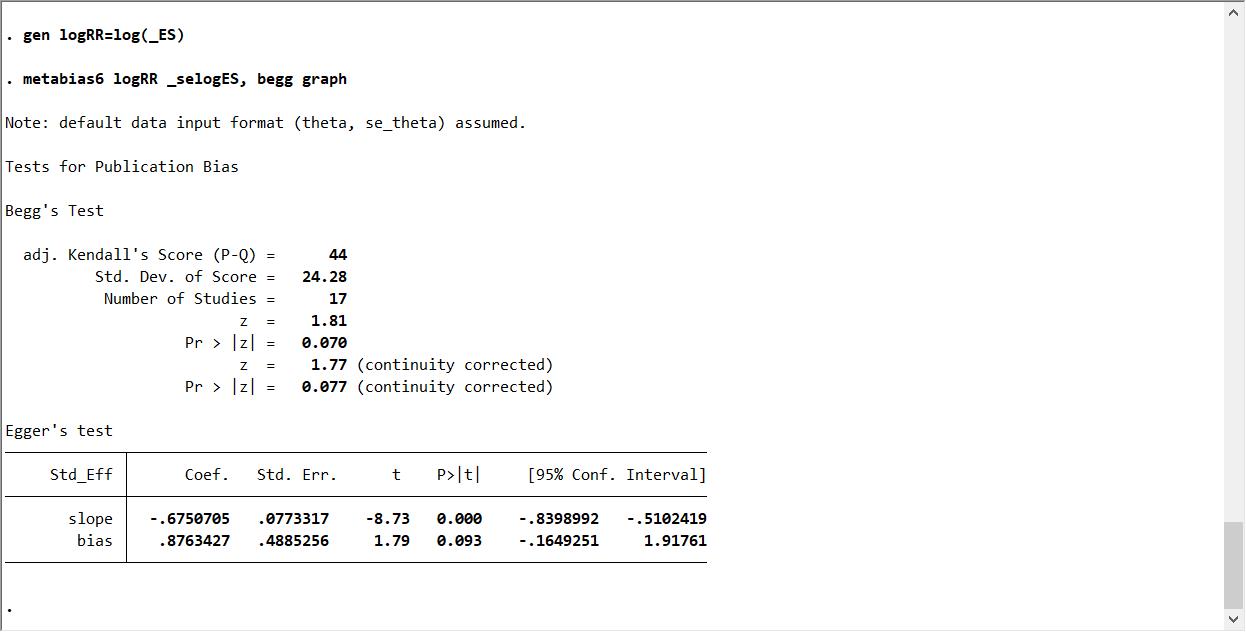

Supplement: Supplementary file 1 [file 2153-8174-24-4-116-s1.zip › publication bias of vascular complication.jpg]

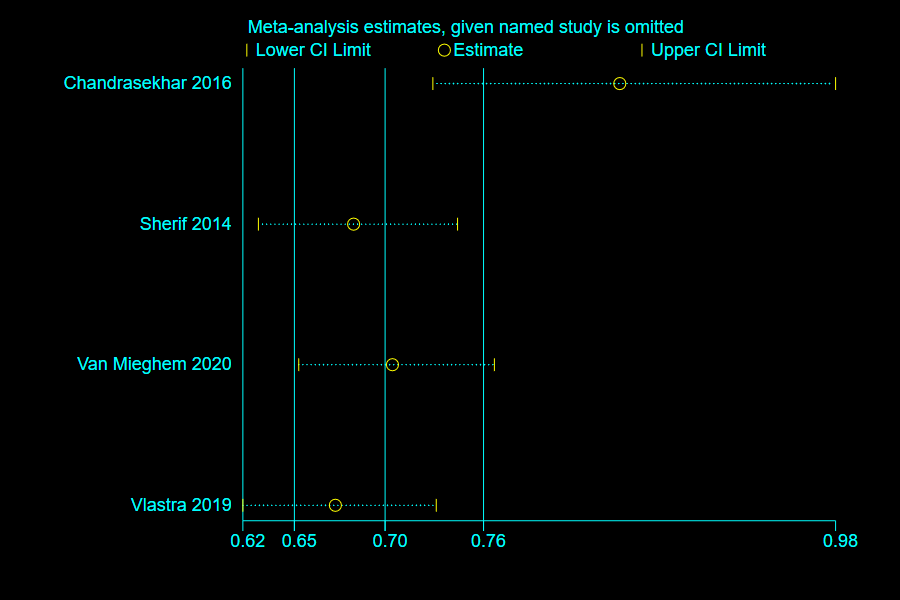

Supplement: Supplementary file 1 [file 2153-8174-24-4-116-s1.zip › sensitivity analysis of atrial fibrillation.tif]

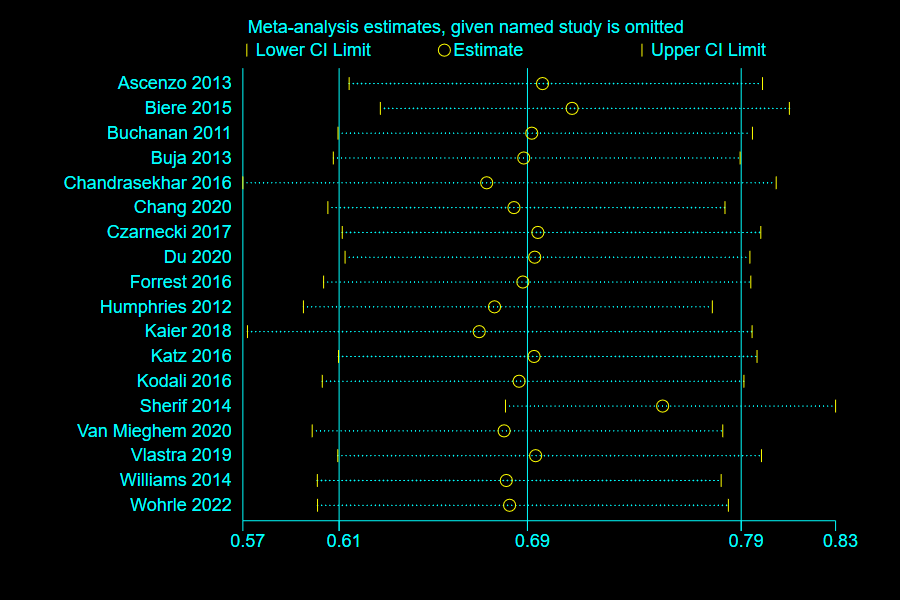

Supplement: Supplementary file 1 [file 2153-8174-24-4-116-s1.zip › sensitivity analysis of bleeding.tif]

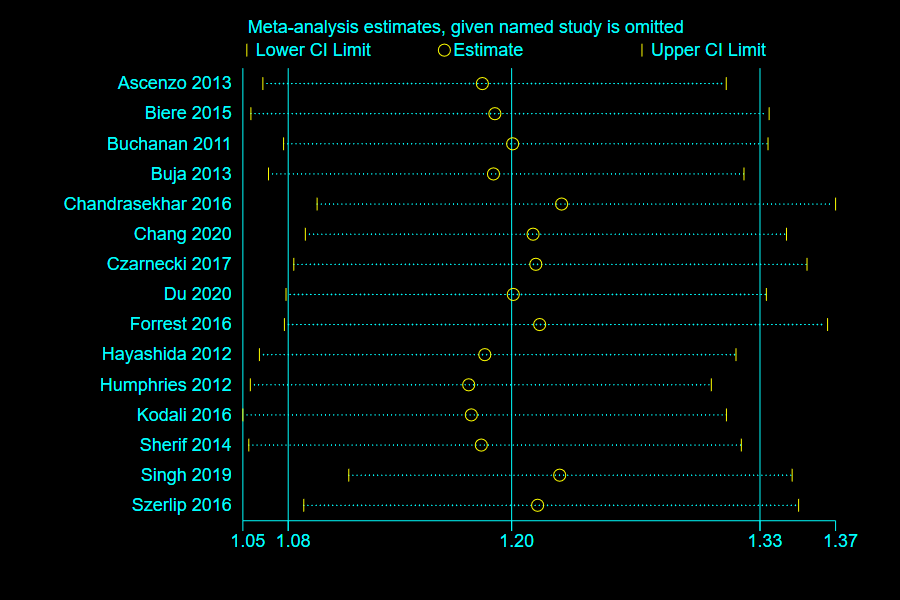

Supplement: Supplementary file 1 [file 2153-8174-24-4-116-s1.zip › sensitivity analysis of one-year mortality.tif]

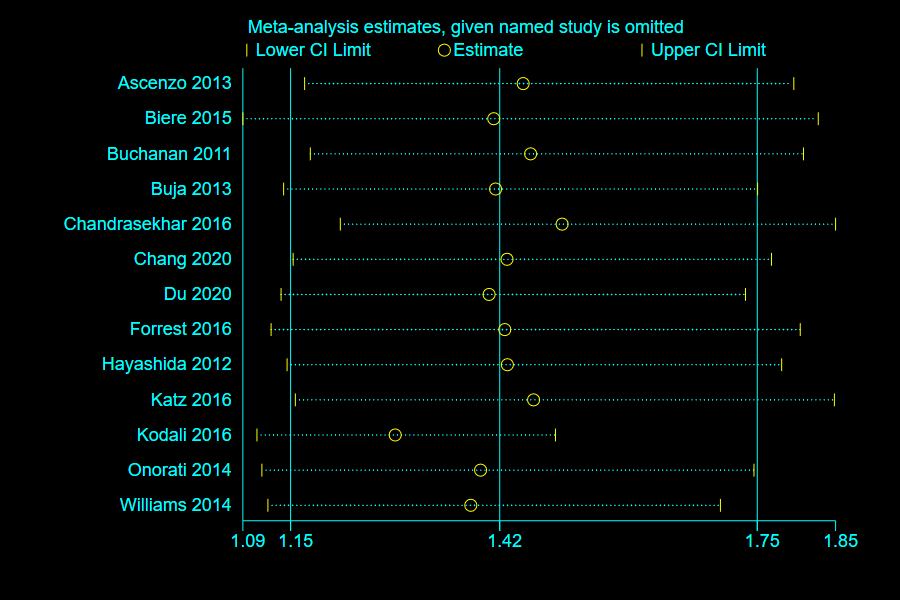

Supplement: Supplementary file 1 [file 2153-8174-24-4-116-s1.zip › sensitivity analysis of PVL.tif]

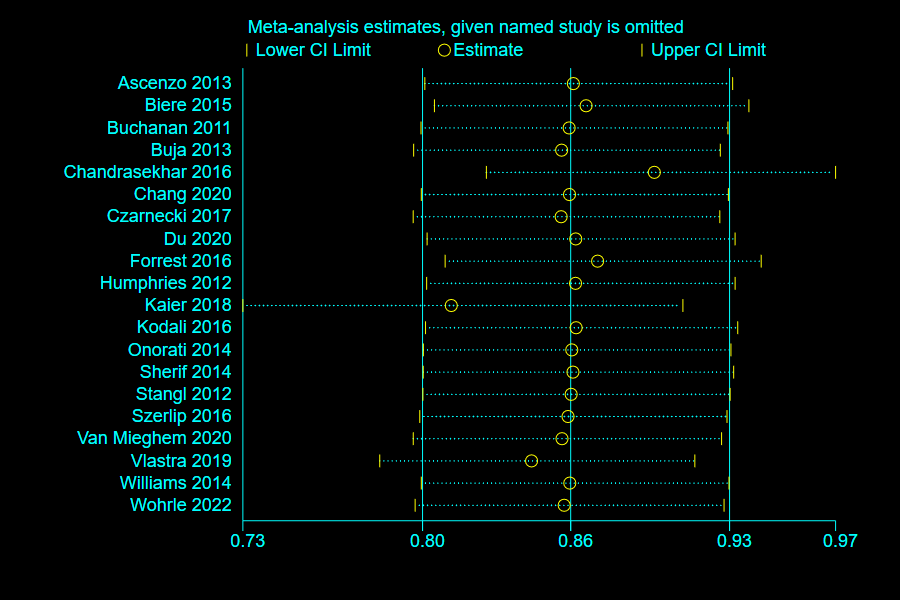

Supplement: Supplementary file 1 [file 2153-8174-24-4-116-s1.zip › sensitivity analysis of stroke.tif]

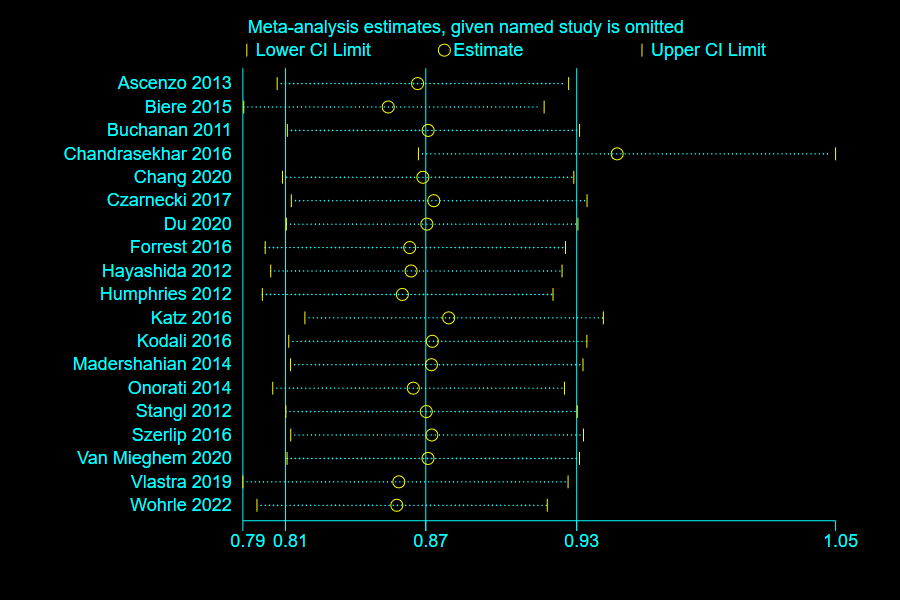

Supplement: Supplementary file 1 [file 2153-8174-24-4-116-s1.zip › sensitivity analysis of thirty-day mortality.tif]

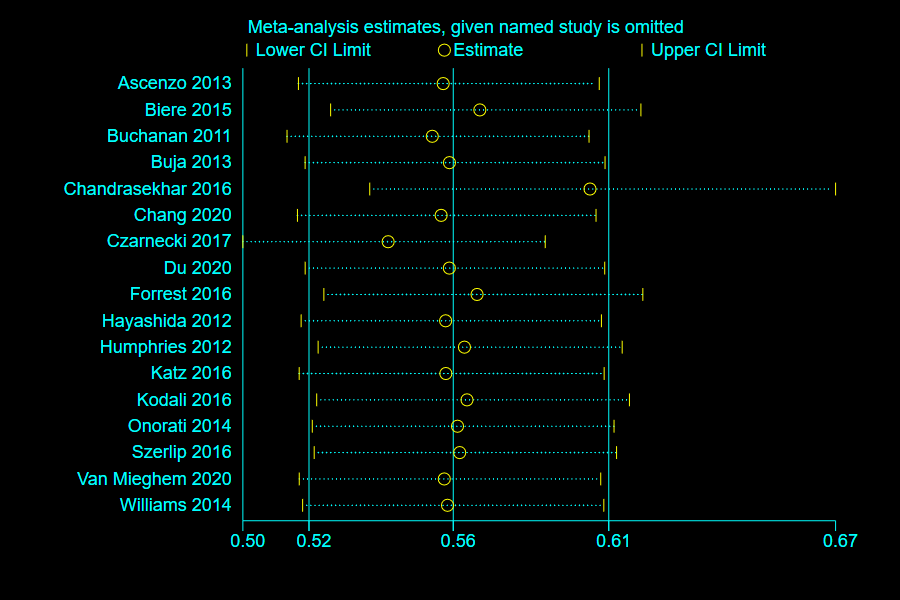

Supplement: Supplementary file 1 [file 2153-8174-24-4-116-s1.zip › sensitivity analysis of vascular complication.tif]

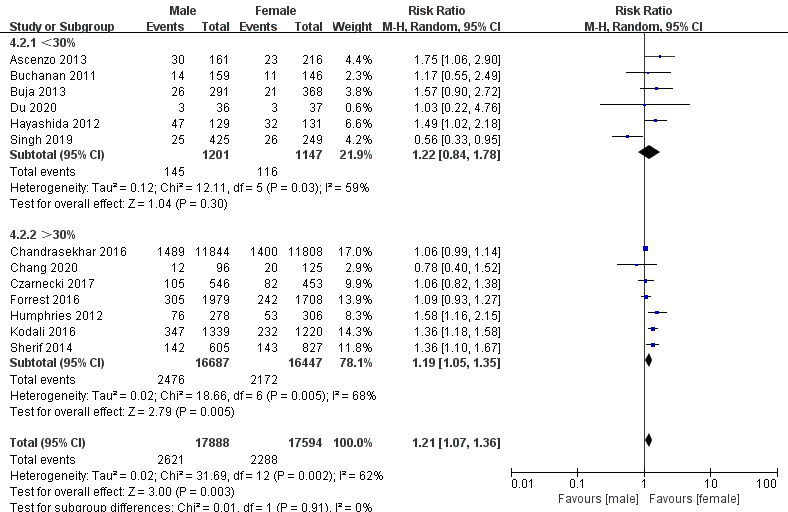

Supplement: Supplementary file 1 [file 2153-8174-24-4-116-s1.zip › Subgroup analysis of DM (1-year mortality).png]

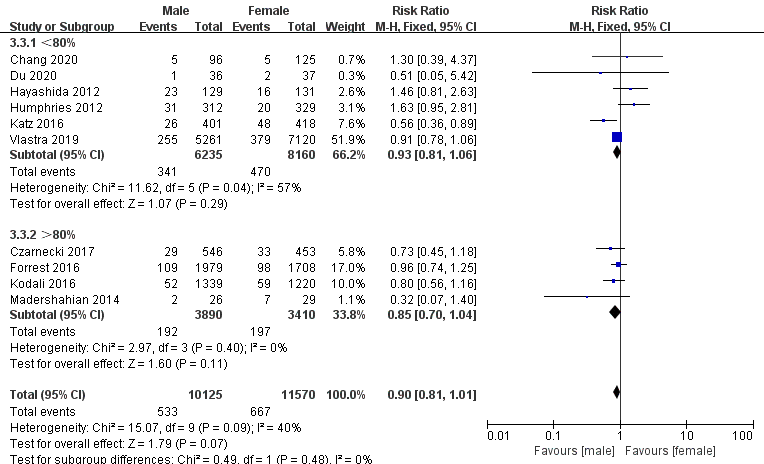

Supplement: Supplementary file 1 [file 2153-8174-24-4-116-s1.zip › subgroup analysis of HT in 1-year mortality.png]

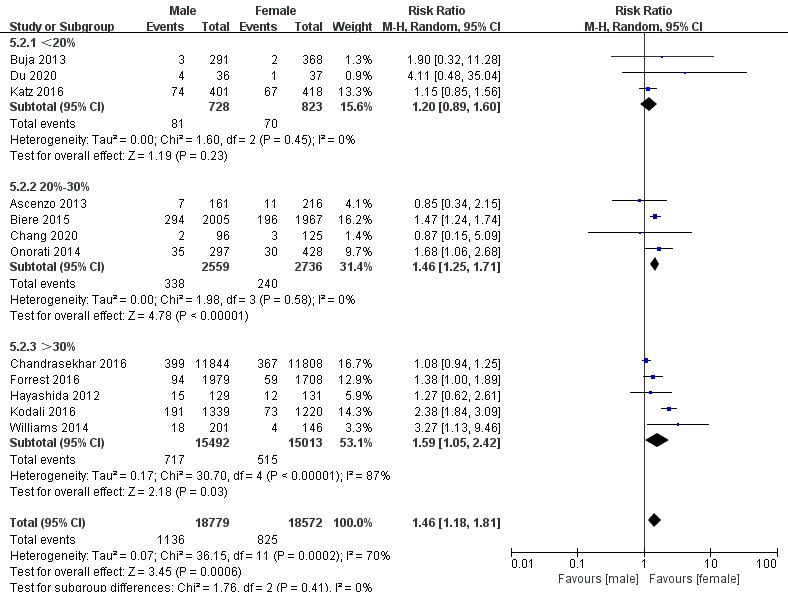

Supplement: Supplementary file 1 [file 2153-8174-24-4-116-s1.zip › subgroup analysis of PAD in PVL.png]
