# Supplementary material for: Influence of Gender on Transcatheter Aortic Valve Implantation: A Systematic Review and Meta-Analysis
Source: Rev Cardiovasc Med. 2023 Apr 18;24(4):116. doi: 10.31083/j.rcm2404116 (PMC11273049; doi:10.31083/j.rcm2404116)
Supplement: Supplementary file 1 [file 2153-8174-24-4-116-s1.zip › search strategy.doc]

(("Transcatheter Aortic Valve Replacement"[Mesh]) OR ("Transcatheter Aortic Valve Replacement"[Title/Abstract]) OR ("Transcatheter Aortic Valve Implantation"[Title/Abstract]) OR ("TAVR"[Title/Abstract]) OR ("TAVI"[Title/Abstract]) ) AND (("aorta"[MeSH])OR ("aorta"[Title/Abstract] )OR ("aortas"[Title/Abstract] )OR ("aortae"[Title/Abstract])OR (("Femoral Artery"[Mesh]) OR ("Femoral Arteries"[Title/Abstract]) OR( "Common Femoral Artery"[Title/Abstract]) OR ("Common Femoral Arteries"[Title/Abstract]) OR(("Internal Carotid Arteries"[MeSH]) OR ("Internal Carotid Arteries"[Title/Abstract])OR("Internal Carotid Artery"[Title/Abstract]) OR (("transapical" [Mesh])OR("transapical"[Title/Abstract]) OR( "apical"[Title/Abstract]) OR( "ventricular apex"[Title/Abstract]) OR( "apex"[Title/Abstract]) OR( "apex cordis"[Title/Abstract]) OR ("cardiac apex"[Title/Abstract]） AND (("Aortic Valve Stenosis"[Mesh]) OR ("Aortic Valve Stenoses"[Title/Abstract] OR "Aortic Stenosis"[Title/Abstract]) OR (("Aortic Valve Insufficiency"[Mesh]) OR ("Aortic Regurgitation"[Title/Abstract]) OR ("Aortic Incompetence"[Title/Abstract]) OR ("Aortic Valve Incompetence"[Title/Abstract]))
